# Optimizing cofactor availability for the production of recombinant heme peroxidase in *Pichia pastoris*

**DOI:** 10.1186/s12934-014-0187-z

**Published:** 2015-01-13

**Authors:** Florian W Krainer, Simona Capone, Martin Jäger, Thomas Vogl, Michaela Gerstmann, Anton Glieder, Christoph Herwig, Oliver Spadiut

**Affiliations:** Graz University of Technology, NAWI Graz, Institute of Molecular Biotechnology, Graz, Austria; Vienna University of Technology, Institute of Chemical Engineering, Research Area Biochemical Engineering, Gumpendorfer Strasse 1a, 1060 Vienna, Austria

**Keywords:** *Pichia pastoris*, Recombinant protein production, Plant peroxidase, Horseradish peroxidase, Metabolic engineering, Cofactor, Heme, Heme biosynthesis, Apo-protein

## Abstract

**Background:**

Insufficient incorporation of heme is considered a central impeding cause in the recombinant production of active heme proteins. Currently, two approaches are commonly taken to overcome this bottleneck; metabolic engineering of the heme biosynthesis pathway in the host organism to enhance intracellular heme production, and supplementation of the growth medium with the desired cofactor or precursors thereof to allow saturation of recombinantly produced apo-forms of the target protein. In this study, we investigated the effect of both, pathway engineering and medium supplementation, to optimize the recombinant production of the heme protein horseradish peroxidase in the yeast *Pichia pastoris*.

**Results:**

In contrast to studies with other hosts, co-overexpression of genes of the endogenous heme biosynthesis pathway did not improve the recombinant production of active heme protein. However, medium supplementation with hemin proved to be an efficient strategy to increase the yield of active enzyme, whereas supplementation with the commonly used precursor 5-aminolevulinic acid did not affect target protein yield.

**Conclusions:**

The yield of active recombinant heme peroxidase from *P. pastoris* can be easily enhanced by supplementation of the cultivation medium with hemin. Thereby, secreted apo-species of the target protein are effectively saturated with cofactor, maximizing the yield of target enzyme activity.

**Electronic supplementary material:**

The online version of this article (doi:10.1186/s12934-014-0187-z) contains supplementary material, which is available to authorized users.

## Background

**Figure 1:**
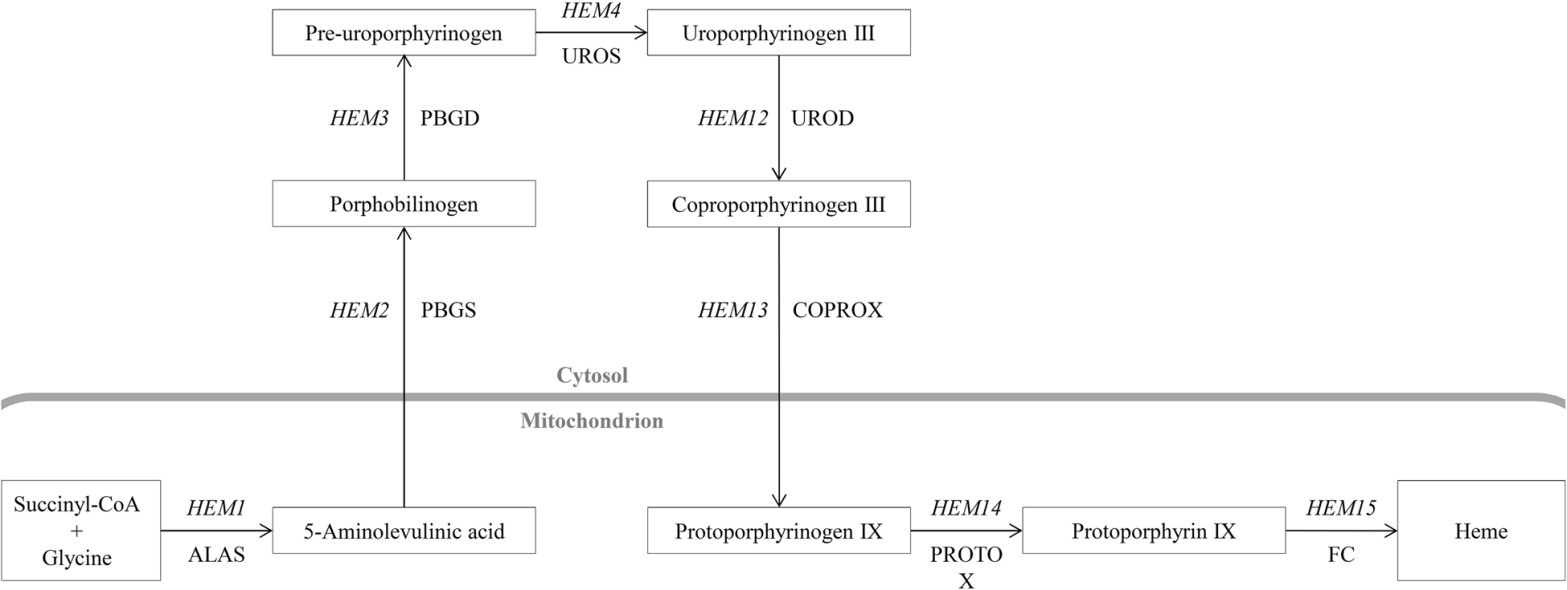
**Scheme of the heme biosynthesis pathway.** ALAS, 5-aminolevulinic acid synthase; PBGS, porphobilinogen synthase; PBGD, porphobilinogen deaminase; UROS, uroporphyrinogen III synthase; UROD, uroporphyrinogen III decarboxylase; COPROX, coproporphyrinogen III oxidase; PROTOX, protoporphyrinogen IX oxidase; FC, ferrochelatase.

The methylotrophic yeast *Pichia pastoris* is a valuable host for the recombinant production of complex proteins. A considerable number of these proteins requires cofactors, amongst others heme, to form active biocatalysts. Heme biosynthesis (Figure 1) is tightly regulated and highly conserved throughout evolution [1-3].

To deepen our understanding on pathway regulation and improve cofactor availability for recombinant heme proteins, metabolic engineering of the heme biosynthesis pathway has been performed in *Aspergillus niger* [4,5], *Escherichia coli* [6] and *Saccharomyces cerevisiae* [7-9]. Despite the high conservation of the heme biosynthesis pathway, distinct differences were found among the different species. In *E. coli*, formation of 5-aminolevulinic acid (ALA) by the *HEM1*-encoded ALA synthase was described as rate-limiting step [10], whereas in *S. cerevisiae* the *HEM2*- and *HEM3*-encoded porphobilinogen synthase and deaminase, respectively, were described as rate-limiting [7]. Based thereon, overexpression of *HEM3* alone or in combination with *HEM2* and *HEM12* was described to be a valuable strategy to augment the production of recombinant heme proteins in *S. cerevisiae* [9,11]. Despite these promising results, there are some potential pitfalls connected to metabolic pathway engineering, such as the additional metabolic burden upon overexpression of multiple genes as well as an accumulation of free intracellular porphyrin intermediates which can lead to the formation of reactive oxygen species [12]. Thus, medium supplementation with iron, heme precursors or hemin (the ferric chloride species of heme) was assessed as alternative strategy to effectively saturate recombinant apo-species of heme proteins [13-17].

In this study, we aimed at optimizing heme availability and thus boost the amount of recombinant active heme protein, namely horseradish peroxidase (HRP), in the yeast *P. pastoris* by evaluating pathway engineering and medium supplementation. In a systematic approach, we co-overexpressed HRP with genes of the endogenous heme biosynthesis pathway of *P. pastoris*, and assessed the effect of medium supplementation with iron, ALA and hemin on the yield of target enzyme activity.

## Results and discussion

### Heme biosynthesis pathway in *Pichia pastoris*

The heme biosynthesis pathway of several organisms has been described before and was found to be highly conserved [1-3]. Based on the *HEM* gene sequences from *S. cerevisiae*, we identified the corresponding homologs in the partially annotated genome of *P. pastoris* [18] *in silico* (Table 1). Sanger sequencing confirmed the correct nucleotide sequences of *HEM1*, *HEM2*, *HEM3*, *HEM4*, *HEM12*, *HEM13* and *HEM14*. For *HEM15*, nucleotide 918 was G in the GenBank database entries of the published genomes of *P. pastoris* strains CBS 7435 and GS155, but T in the Sanger-sequenced CBS 7435 *P. pastoris* strain used in the present study (Additional file 1). However, this single nucleotide polymorphism only resulted in a silent mutation and did not alter the amino acid sequence of the encoded protoheme ferro-lyase.

**Table 1:** ***In silico*** **identification of** ***HEM*** **homologs in** ***P. pastoris***

***S. cerevisiae***	***P. pastoris***			**Sequence identity [%]**
**Gene annotation**	**Gene annotation**	**Chromosome**	**COG annotation**	
*HEM1*	*HEM1*	II	5-aminolevulinate synthase	67.7
*HEM2*	*HEM2*	IV	delta-aminolevulinic acid dehydratase	75.3
*HEM3*	*HEM3*	I	porphobilinogen deaminase	54.0
*HEM4*	*HEM4*	II	uroporphyrinogen-III synthase	42.3
*HEM12*	n/a	III	uroporphyrinogen decarboxylase	73.2
*HEM13*	*HEM13*	III	coproporphyrinogen III oxidase	65.5
*HEM14*	n/a	IV	protoporphyrinogen oxidase	33.7
*HEM15*	n/a	III	protoheme ferro-lyase (ferrochelatase)	61.2

Genes *HEM12*, *HEM14* and *HEM15* were not annotated (n/a) for *P. pastoris* CBS 7435. Their chromosomal position in the genome, eukaryotic cluster of orthologous groups (COG; [19]) annotations and identities of the encoded amino acid sequences from *S. cerevisiae* and *P. pastoris* are shown.

### Co-overexpression of *HEM* genes in microscale cultivations

**Figure 2:**
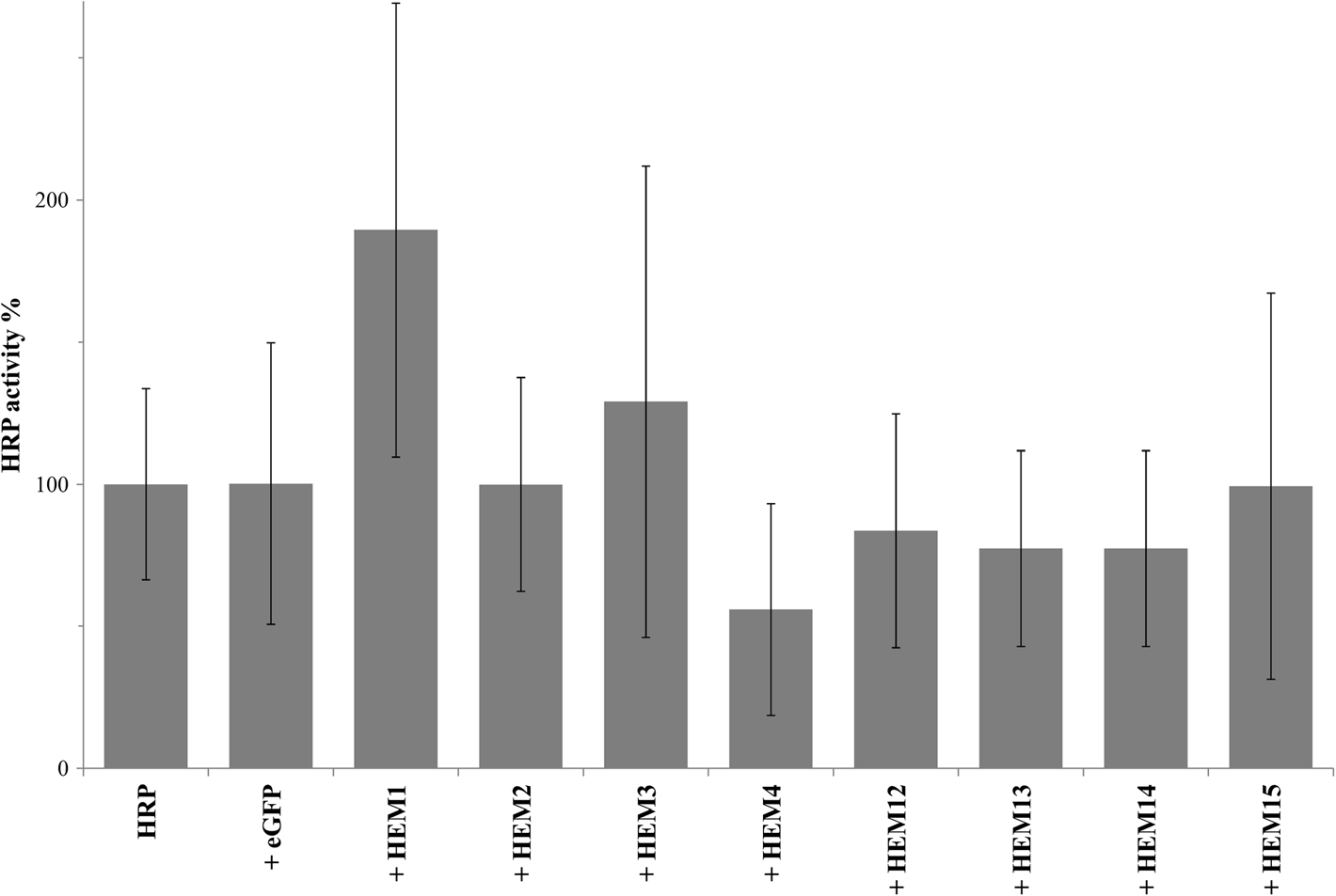
**Co-overexpressions of** ***HEM*** **genes with HRP.** HRP overexpression was regulated by P*AOX1*, co-overexpressions of eGFP and *HEM* were regulated by P*GAP*. Bars are average values of HRP production landscapes from microscale cultivations using 1 mM FeSO_4_-supplemented medium. Error bars are standard deviations from all measured clones of a landscape. Average activity from strains producing HRP alone was set to 100%.

In a recent study it was shown that metabolic engineering of the heme biosynthesis pathway allowed higher yields of active recombinant heme protein in *S. cerevisiae* [11]. Thus, we co-overexpressed either of the eight involved *HEM* genes (Table 1) using the strong constitutive promoter P*GAP* [20] in a *P. pastoris* strain recombinantly producing the heme protein HRP. The screenings revealed trends of co-overexpressed *HEM1* and *HEM3* to be potentially beneficial for the production of active HRP (Figure 2). Surprisingly, there also seemed to be a negative trend upon co-overexpression of *HEM4*. We hypothesize, that *HEM4* co-overexpression might have led to the accumulation of a cytotoxic intermediate, which increased intracellular stress and ultimately caused a disadvantageous production environment for HRP under the tested conditions. At this point, we did not follow up on the effects of *HEM4* co-overexpression. Since co-overexpression of eGFP as negative control did not affect HRP productivity, the observed activity-enhancing trends seen for *HEM1* and *HEM3* were considered a consequence of the co-overexpressed *HEM* gene, although the standard deviation in these experiments was high. The high standard deviations in this screening (Figure 2) were caused by the transformant-to-transformant variation typically observed for *P. pastoris* [21]. Even the transformation of a single gene results in transformants showing different expression strengths. The majority of the transformants shaping the landscape behaved similar but a few transformants showed either no expression or elevated levels, leading to high standard deviations. Supplementation of the cultivation medium with 1 mM FeSO_4_ to alleviate a potential iron limitation gave comparable results to non-supplemented medium and did not affect the observed trends upon *HEM* gene co-overexpression (data not shown).

In addition to strong constitutive co-overexpression of either *HEM1* or *HEM3* from P*GAP*, we also tested co-overexpression of these two genes from either P*AOX1* or P*CAT1*. Both promoters are strongly methanol-inducible, however P*CAT1* is already active in the absence of glucose or glycerol and then even further induced by addition of methanol, thereby allowing *HEM* co-overexpression already prior to, but also during HRP production [Vogl *et al.*, manuscript in preparation]. In order to indirectly assess the general functionality and applicability of the employed co-overexpression construct, we measured fluorescence of the eGFP co-overexpression control transformants and found this co-overexpression partner to be successfully produced (Additional file 2). Thus, we considered the endogenous *HEM* co-overexpression partners to be co-overexpressed in a comparable fashion. Ultimately, P*AOX1*-regulated co-overexpressions of *HEM1* and *HEM3* with HRP were found most promising (Additional file 3) and the best performing strains were used for further characterizations.

Since the data from microscale cultivations indicated merely trends for potential beneficial effects of co-overexpressed *HEM1* or *HEM3*, we aimed at obtaining more reliable data from controlled cultivations of the best performing strains in bioreactors. Prior to bioreactor cultivations, the strain producing HRP alone (hereafter called benchmark strain) as well as the *HEM1* and *HEM3* co-overexpressing strains (hereafter called HEM1 and HEM3 strains) were characterized by quantitative real-time PCR as strains with single copy integration of the HRP gene. Since these strains contained the same HRP gene dosage, genomic rearrangement of HRP gene copy number upon transformation of a *HEM* co-overexpression construct was excluded and the strains were regarded comparable in terms of target gene dosage.

### Co-overexpression of *HEM* genes in bioreactor cultivations

For physiological strain characterisation of the three yeast strains we employed a previously reported strategy of dynamic batch cultivations with methanol pulses [22-25]. The corresponding off-gas signals, specific uptake rates and yields are shown in Additional file 4. Physiological strain characteristic parameters of the three strains are summarized in Table 2. As shown in Table 2, C-balances for all cultivations closed to 1.0 indicating solid data quality. Apparently, the yeast strains were physiologically not impaired by co-overexpression of either *HEM1* or *HEM3* since adaptation time to methanol, yields and specific substrate uptake rates were comparable. However, co-overexpressing these genes did not boost the production of active HRP either. Whereas the benchmark and the HEM1 strain showed comparable production titres, the HEM3 strain even showed a 50% lower specific and volumetric productivity than the benchmark strain. Taken together, it turned out that the trends seen in microscale data could not be seen in the bioreactor. A possible explanation for these diverging findings could be found in the considerably different cultivation conditions. Microscale cultivations may challenge cell growth and productivity by phases of O_2_ shortage or starvation, whereas a bioreactor provides optimal cultivation conditions.

**Table 2:** **Strain characteristic physiological parameters determined for the benchmark, HEM1 and HEM3 strain**

**Strain**	**Δtime**_**adapt**_ **[h]**	**Y**_**X/MeOH**_ **[C-mol/C-mol]**	**Y**_**CO2/MeOH**_ **[C-mol/C-mol]**	**C-balance**	**q**_**MeOH**_ **[mmol/g/h]**	**q**_**p**_ **[U/g/h]**	**r**_**p**_ **[U/L/h]**
Benchmark	8.2	0.02	1.00	1.02	0.75	1.11	37.5
HEM1	8.2	0.04	0.94	0.98	0.69	1.10	35.9
HEM3	7.9	0.03	0.93	0.96	0.70	0.49	17.1

Δtime_adapt_, time for adaptation of the culture to methanol; Y_X/MeOH_, Y_CO2/MeOH_, yields of biomass or CO_2_ per C-mol of substrate methanol; C-balance, sum of Y_X/MeOH_ and Y_CO2/MeOH_ which ideally should result in 1.0; q_MeOH_, average specific uptake rate of methanol during consecutive methanol pulses; q_p_, specific HRP productivity; r_p_, volumetric HRP productivity calculated from the point of induction until the end of cultivation.

Considering the complex and so far poorly understood regulation of heme biosynthesis, a multitude of single or combined factors may have either positive or negative effects on this pathway. Thus, additional studies will be required to unravel the regulation of heme biosynthesis, in order to allow non-speculative conclusions. Since our selection of *HEM* co-overexpression partners was based on trends from microscale cultivations, we cannot exclude, that either of the remaining six *HEM* genes or combinations thereof might yield more beneficial results than co-overexpressed *HEM1* or *HEM3* in bioreactor cultivations. Ultimately, in contrast to analogous studies in *S. cerevisiae* [9,11], metabolic pathway engineering in *P. pastoris* did not prove to be a useful strategy to allow higher titres of recombinant active heme protein, suggesting distinct differences of this pathway between the two yeasts. However, co-overexpression of two or more *HEM* genes from a library of promoters of varying strengths might still enhance endogenous heme biosynthesis in *P. pastoris* and will be assessed in future studies.

### Medium supplementation

#### Medium supplementation in microscale cultivations

**Figure 3:**
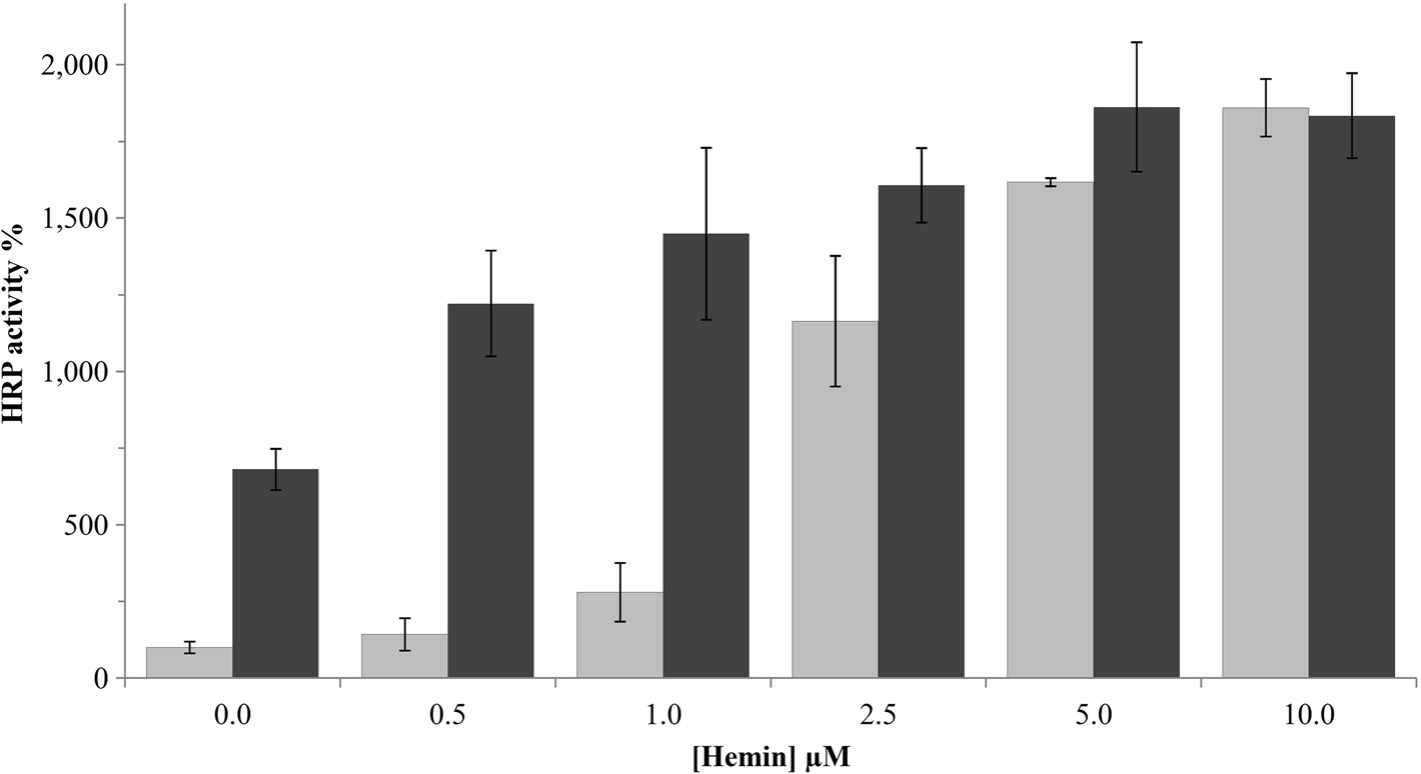
**Effect of medium supplementation with FeSO**_**4**_ **and hemin on HRP activity in microscale cultivations.** Volumetric HRP activity from minimal medium without supplementation was set to 100%. Bars are average values ± SD from independent triplicate cultivations. Light gray, no FeSO_4_; dark gray, 1 mM FeSO_4_.

We tested ALA, FeSO_4_ and hemin as medium supplements in microscale cultivations of the benchmark strain. Supplementation with ALA at an excess concentration of 1 mM, as reported in literature [22,26,27], did not result in measurably more active product. However, addition of 1 mM FeSO_4_ boosted the amount of active HRP up to 7-fold (Figure 3), hinting at a potential shortage of iron in heme biosynthesis. Upon supplementation of the minimal medium with different concentrations of hemin, we even measured 18-fold increased HRP activity compared to non-supplemented conditions. We also investigated the effect in case both, FeSO_4_ and hemin, were supplemented concomitantly and observed that the beneficial effect of FeSO_4_ became less pronounced with increasing concentrations of hemin (Figure 3). Apparently, as all apo-HRP was readily saturated with cofactor at a hemin concentration of 10 μM, additional excess of iron did not improve HRP activities any further and the cofactor bottleneck was opened up.

#### ALA supplementation in the bioreactor

We performed a comparative cultivation experiment in the bioreactor to confirm the results obtained in microscale cultivations that the commonly used supplement ALA [22,26,27] does not affect the production of a recombinant active heme protein in *P. pastoris*. We cultivated the benchmark strain in parallel dynamic batch cultivations with and without the presence of 1 mM ALA. Table 3 shows that both, strain physiology and productivity, were not affected by the presence of ALA. Since the amount of active HRP was the same for both conditions, we concluded that ALA was not beneficial for the activation of the produced apo-HRP. This observation is different from what was described previously by Morawski *et al*., where a 32% increase in HRP activity was measured in the culture supernatant after supplementation with ALA, trace elements and thiamine [17]. Thus, we speculate that the activation effect observed by Morawski *et al*. was rather caused by the addition of the iron-containing trace element solution than by ALA. Furthermore, our results conclusively showed that the intracellular production of ALA in the heme biosynthesis pathway of *P. pastoris* was not the rate limiting step in contrast to *E. coli* [10], which is also in agreement with the study of Arrese *et al.* [28].

**Table 3:** **Strain specific physiological parameters of the benchmark strain with and without ALA supplementation**

**ALA [1 mM]**	**Δtime**_**adapt**_ **[h]**	**Y**_**X/MeOH**_ **[C-mol/C-mol]**	**Y**_**CO2/MeOH**_ **[C-mol/C-mol]**	**C-balance**	**q**_**MeOH**_ **[mmol/g/h]**	**q**_**p**_ **[U/g/h]**	**r**_**p**_ **[U/L/h]**
+	8.1	0.03	0.95	0.98	0.73	1.08	36.7
-	8.2	0.04	0.94	0.98	0.76	1.09	37.2

Δtime_adapt_, time for adaptation of the culture to methanol; Y_X/MeOH_, Y_CO2/MeOH_, yields of biomass or CO_2_ per C-mol of substrate methanol; C-balance, sum of Y_X/MeOH_ and Y_CO2/MeOH_ which ideally should result in 1.0; q_MeOH_, average specific uptake rate of methanol during consecutive methanol pulses; q_p_, specific HRP productivity; r_p_, volumetric HRP productivity calculated from the point of induction until the end of cultivation.

#### Optimization of hemin supplementation in the bioreactor

Based on the results obtained in microscale cultivations, we supplemented the cultivation broth of three parallel dynamic batch cultivations of the benchmark strain with 0.1, 1.0 or 10.0 μM hemin. As shown in Table 4, adaptation time, yields and uptake rates of the benchmark strain were not affected by the presence of hemin. In terms of productivity, both the specific and the volumetric productivity were doubled at a concentration of 10.0 μM hemin. Since the total extracellular protein content was comparable in all three cultivations, we concluded that not the productivity of the yeast was altered, but rather that the produced apo-protein was activated posttranslationally by hemin in the cultivation broth. Based on our previous experiences with this expression system [22,23,29,30], we estimated the amount of HRP in the cultivation broth to be < 3 μM. Thus, we concluded that an excess hemin concentration is required to effectively saturate secreted apo-HRP (Table 4).

**Table 4:** **Strain characteristic physiological parameters determined for the benchmark strain cultivated in hemin-supplemented media**

**Hemin [μM]**	**Δtime**_**adapt**_ **[h]**	**Y**_**X/MeOH**_ **[C-mol/C-mol]**	**Y**_**CO2/MeOH**_ **[C-mol/C-mol]**	**C-balance**	**q**_**MeOH**_ **[mmol/g/h]**	**q**_**p**_ **[U/g/h]**	**r**_**p**_ **[U/L/h]**
0.1	6.5	0.04	0.89	0.93	0.69	1.10	34.3
1.0	6.4	0.04	0.93	0.97	0.71	1.26	36.8
10.0	6.5	0.03	0.89	0.96	0.70	2.35	73.2

Δtime_adapt_, time for adaptation of the culture to methanol; Y_X/MeOH_, Y_CO2/MeOH_, yields of biomass or CO_2_ per C-mol of substrate methanol; C-balance, sum of Y_X/MeOH_ and Y_CO2/MeOH_ which ideally should result in 1.0; q_MeOH_, average specific uptake rate of methanol during consecutive methanol pulses; q_p_, specific HRP productivity; r_p_, volumetric HRP productivity.

### Activation studies with hemin

To prove our hypothesis of a posttranslational conversion of apo-HRP to holo-HRP by hemin, we incubated cell-free, sterile-filtered supernatant from a non-supplemented benchmark cultivation with different concentrations of hemin and analyzed activity over time. As shown in Table 5, already after 5 min of incubation a concentration dependent activation was observed and after 72 h of incubation the initial activity was even doubled. Based thereon, we concluded that hemin should be present in excess to allow effective apo-protein activation.

**Table 5:** **Posttranslational activation of apo-HRP with hemin**

**Hemin [μM]**	**Specific activity [U/mg] after 5 min of incubation**	**Specific activity [U/mg] after 72 h of incubation**
-	34.8	35.3
1.0	42.4	67.0
5.0	46.8	75.4
10.0	51.8	77.8

Cell-free cultivation supernatant was incubated with the indicated concentrations of hemin and volumetric HRP activity was measured after 5 min and 72 h.

## Conclusions

In this study we present a systematic approach to optimize heme availability and thus boost the amount of active heme protein produced in the yeast *P. pastoris* by evaluating metabolic pathway engineering and medium supplementation. The results can be summarized as:

- The heme biosynthesis pathway of *P. pastoris* was analyzed and corresponding genes were identified and annotated *in silico*.
- In contrast to previous studies, overexpression of single *HEM* genes did not result in enhanced activity or higher yield of the model heme protein HRP in bioreactor cultivations of recombinant *P. pastoris* strains. However, combinations of *HEM* genes co-overexpressed from a library of differently regulated promoters might still enhance endogenous heme biosynthesis of *P. pastoris*.
- Medium supplementation with the traditionally used and pricy heme precursor ALA did not increase the yield of active product and can be omitted in future cultivations. FeSO_4_ and hemin on the other hand turned out to be useful medium supplements to increase the yield of active heme protein.
- Hemin was identified as the most effective supplement. It activated the secreted model heme protein posttranslationally in the cultivation broth and should be added in moderate excess to effectively saturate secreted apo-species of the target protein.

The results shown in this study present a guideline for the successful recombinant production of active heme protein in the yeast *P. pastoris* and offer an easy-to-do solution to maximize the ratio of holo- over apo-protein resulting in a considerable increase of active target protein.

## Methods

### Chemicals

2,2′-azino-bis(3-ethylbenzthiazoline-6-sulfonic acid) diammonium salt (ABTS), D(+)-biotin and hemin were purchased from Sigma-Aldrich (Austria). Difco™ yeast nitrogen base (YNB) w/o amino acids, Bacto™ tryptone and Bacto™ yeast extract were obtained from Becton Dickinson (Austria). Zeocin™ was obtained from InvivoGen (France). Other chemicals were obtained from Carl Roth (Germany).

#### *Pichia pastoris* strains

All strains in this study were based on the *P. pastoris* wildtype strain CBS 7435 (identical to NRRL Y-11430 and ATCC 76273). The Mut^S^ phenotype has been found previously to be superior over the Mut^+^ phenotype for recombinant HRP production in terms of volumetric productivity and production efficiency [24]. Hence, an *aox1* deletion strain (*Pp*MutS) was used as starting strain [31]. Homologs of the *HEM* genes of *S. cerevisiae* were identified by BLAST searches [32] in the published genome sequence of *P. pastoris* CBS 7435 [18] and the sequences were verified by Sanger sequencing (Microsynth, Austria). Sequence identities of the *HEM* gene products from *S. cerevisiae* and *P. pastoris* were determined on the LALIGN server.

The HRP expression construct was based on the pPpT4_Alpha_S vector [31] where HRP C1A expression was under control of the promoter of the *AOX1* gene (P*AOX1*). Transformants were identified by Zeocin™ resistance. The *S. cerevisiae* MATα prepro signal peptide facilitated HRP secretion. All *HEM* co-overexpression constructs were based on the plasmid pPpKan_S [32], harboring a kanamycin resistance gene for selection and the strongly methanol-inducible P*AOX1* for gene expression. Also, the promoters P*GAP* and P*CAT1* [Vogl *et al*., manuscript in preparation] were tested for co-overexpression. Transformation of the HRP expression cassette to *Pp*MutS and of the co-overexpression constructs to the *Pp*MutS-based HRP production strain (called benchmark strain) was performed as described previously [24]. All eight *HEM* genes of the heme biosynthesis pathway were co-overexpressed separately and their influence on HRP production was studied by cultivation of approximately 80 transformants per co-overexpression construct in 96-deep well plates (DWP). As negative co-overexpression control, eGFP was co-overexpressed with HRP. The copy number of the HRP gene in selected strains was determined by quantitative real-time PCR according to a previous protocol [33] and as described recently [24].

### Microscale cultivations in 96-deep well plates

Microscale cultivations in 96-DWPs were performed similar to [34]: Strains were grown in 250 μL BMD1% (11 g/L alpha-D(+)-glucose monohydrate, 13.4 g/L YNB, 0.4 mg/L D(+)-biotin, 0.1 M potassium phosphate buffer, pH 6.0) at 28°C, 320 rpm, 80% humidity. After approximately 60 h, an induction pulse of 250 μL BMM2 (1% (v/v) methanol, 13.4 g/L YNB, 0.4 mg/L D(+)-biotin, 0.1 M potassium phosphate buffer, pH 6.0) was added, followed by three pulses of 50 μL BMM10 (5% (v/v) methanol, 13.4 g/L YNB, 0.4 mg/L D(+)-biotin, 0.1 M potassium phosphate buffer, pH 6.0) per well 12, 24 and 36 h after the first pulse. HRP activity was determined by mixing 15 μL supernatant with 140 μL assay solution (1 mM ABTS, 0.9 mM H_2_O_2_, 50 mM sodium acetate, pH 4.5) in a microtiter plate and following the increase in absorbance at 405 nm on a Spectramax Plus 384 platereader (MolecularDevices, Germany) at room temperature. Medium supplementation studies were performed by additions of ALA, FeSO_4_ and hemin to BMD1% from a 100 mM ALA stock, a 100 mM FeSO_4_ stock and a 500 μM hemin stock (in 10 mM KOH), respectively.

### Bioreactor cultivations

#### Preculture

Precultures were grown in YNB medium (0.1 M potassium phosphate buffer, pH 6.0; 3.4 g/L YNB w/o amino acids and ammonium sulfate; 10 g/L (NH_4_)_2_SO_4_; 400 mg/L biotin; 20 g/L glucose) in 1 L shake flasks at 30°C and 230 rpm for 24 h.

#### Dynamic batch cultivations

For dynamic batch cultivations, 1.8 L of double concentrated basal salt medium (BSM; 26.7 mL/L 85% phosphoric acid; 1.17 g/L CaSO_4_ · 2H_2_O; 18.2 g/L K_2_SO_4_; 14.9 g/L MgSO_4_ · 7H_2_O; 4.13 g/L KOH; 0.3 mL/L Antifoam Struktol J650; 60 g/L glycerol) were sterilized in 3 L bioreactor vessels (DR03F; DASGIP, Switzerland). After sterilization, 4.35 mL/L trace element solution (PTM1; 6.0 g/L CuSO_4_ · 5H_2_O; 0.08 g/L NaI; 3.0 g/L MnSO_4_ · H_2_O; 0.2 g/L Na_2_MoO_4_ · 2H_2_O; 0.02 g/L H_3_BO_3_; 0.5 g/L CoCl_2_; 20.0 g/L ZnCl_2_; 65.0 g/L FeSO_4_ · 7H_2_O; 0.2 g/L biotin; 5 mL/L H_2_SO_4_) were added and pH was set to 5.0 with concentrated ammonia solution. The precultures were transferred aseptically to the respective vessel (the inoculation volume was 10%) and a batch phase on glycerol was carried out at 30°C with the stirrer fixed at 900 rpm. Aeration with compressed dry air was set to 1 vvm. Dissolved oxygen (dO_2_) was measured with a sterilizable VisiFerm DO 225 probe (Hamilton, Switzerland) and controlled to be higher than 20%. The pH was measured with a sterilizable electrode (Mettler Toledo, Switzerland) and maintained constant at pH 5.0. Reactor weight was continuously recorded by a precision balance (Sartorius, Germany). Following complete glycerol consumption as indicated by an increase in the offgas signal, temperature was lowered to 25°C and an adaptation pulse of 0.5% (v/v) methanol (containing 4.35 mL/L PTM1) was added. After adaptation, 1.0% (v/v) methanol was pulsed repeatedly (for an example see Additional file 4). Before and after each pulse samples were taken and analyzed for OD_600_, dry cell weight, HRP activity, extracellular protein content and methanol concentration.

### Analysis of growth and expression parameters

Dry cell weight (DCW) was determined by centrifugation of 5 mL fermentation broth (4500 rpm, 10 min, 4°C), washing the pellet with 5 mL water and subsequent drying to a constant weight at 105°C. HRP activity in the cell-free supernatant was determined using a previously described assay [23] in a CuBiAn-XC enzymatic robot (OptoCell, Germany). Protein concentration was determined using a Bradford protein assay kit (Thermo Scientific, USA). All growth and protein expression parameters were determined in duplicates.

### Analysis of substrate concentration

Methanol concentration in the cell-free supernatant was determined by HPLC as described previously [22]. Glycerol concentration was measured from cell-free samples in the CuBiAn-XC enzymatic robot. The device was calibrated with water-diluted glycerol standards ranging from 0 to 0.25 g/L. Samples with higher glycerol concentrations were automatically diluted by the system.

### Calculation of strain physiological characteristics

Physiologically relevant parameters for characterization of the different yeast strains cultivated at different conditions and for quantification of the bioprocess were: Carbon dioxide evolution rate (CER; mmol/L/h), biomass yield (Y_X/MeOH_; C-mol/C-mol), carbon dioxide yield (Y_CO2/MeOH_; C-mol/C-mol), C-balance, specific methanol uptake rate (q_MeOH_; mmol/g/h), specific productivity (q_p_; U/g/h), volumetric productivity (r_p_; U/L/h), specific activity (U/mg). All details on the calculation of these parameters have been published previously [34].

## Additional files

Additional file 1:
***Pichia pastoris*** **CBS 7435** ***HEM*** **open reading frames.** The single nucleotide polymorphism of *HEM15*, T918, is marked in grey. Additional file 2:
**Co-overexpression of eGFP from P** ***AOX1*** **.** The principial applicability of the employed co-overexpression construct is demonstrated indirectly by eGFP fluorescence of the strains co-overexpressing HRP and eGFP. The first two bars represent the starting strain, *Pp*MutS, and the benchmark strain as controls. Fluorescence was determined at ex/em 488/507 nm, and normalized to the OD600 to account for potential growth differences. Additional file 3:
**Co-overexpressions of HRP with either** ***HEM1*** **or** ***HEM3*** **from either P** ***AOX1*** **or P** ***CAT1*** **.** 1, co-overexpression of *HEM1* or *HEM3* from P*AOX1*; 2, co-overexpression of *HEM1* or *HEM3* from P*CAT1.* White bars, eGFP co-overexpression control; black bars, *HEM1* co-overexpression; gray bars, *HEM3* co-overexpression. The benchmark strain was included in all cultivations and set to 100% as reference (first bars). Additional file 4:
**Batch cultivations of three recombinant** ***P. pastoris*** **strains.** 1, benchmark strain; 2, HEM1 strain; 3, HEM3 strain; a/c/e, CER signal (solid line) and specific methanol uptake rate (open circles); b/d/f, carbon dioxide yields (Y_CO2/S_; grey squares) and biomass yields (Y_X/S_; black circles).

## Abbreviations

ABTS: 2,2′’-azino-bis(3-ethylbenzthiazoline-6-sulfonic acid) diammonium salt
ALA: 5-aminolevulinic acid
*AOX1*: Alcohol oxidase 1 gene
CER: Carbon evolution rate
96-DWP: 96-deep well plate
eGFP: Enhanced green fluorescent protein
HRP: Horseradish peroxidase
YNB: Difco™ yeast nitrogen base
