# Supplementary material for: Optimizing cofactor availability for the production of recombinant heme peroxidase in Pichia pastoris
Source: Microb Cell Fact. 2015 Jan 13;14:4. doi: 10.1186/s12934-014-0187-z (PMC4299804; doi:10.1186/s12934-014-0187-z)
Supplement: Additional file 1: — Pichia pastoris CBS 7435 HEM open reading frames. The single nucleotide polymorphism of HEM15, T918, is marked in grey. [file 12934_2014_187_MOESM1_ESM.doc]

**>PpHEM1**

ATGGAGTTTGTCGCCCGTCAGTCCATGAATGCCTGTCCCTTTGTCAGGTCAACTTCTACCCACCATTTGAAGAAGTTGGCAGCAAACAGTTCTCTAGCTGCTACTGCTAGTCATTGTCCCGTGGTTGGCCCTGCTCTCCAACAGCAGAGATACTACTCTCAACCTTCCAAGCCAGCCCAAGCCCAAACCTCCGACATTGCTACTGGGATCAAGAAGGATGTTTCTCCGATCCGTATGGACTCTAATGAAACCGCCTTTGATTACAATGGAATGTATGAGTCTGATCTTGCGAATAAACGTAAAGATAACTCGTATCGTTATTTCAATAACATCAACCGTCTAGCCAAGGAGTTTCCCAAGGCACATCGCCAGACCGAAGATGACAAGGTGACCGTCTGGTGCTCTAACGACTACTTAGGAATGGGTAGGCATCCTGAGATTATCAAAACCATGAAGGCTACCATGGACAAGTACGGTTCCGGAGCAGGAGGAACTAGGAACATTGCAGGTCATAACCACGCCGCTATCAATTTGGAAAGCGAGTTGGCTTGCTTGAACAAGAAGGAAGCGGCTCTGGTGTTTTCATCATGTTTCATAGCTAACGATGCAATCATCTCGTTGTTGGGACAAAAAATCAAAAATTTGGTCATTTTCTCTGACCAGTCGAATCATGCTTCCATGATATTGGGTGTGCGTAACTCCAAAGCGAAGAAGCACATCTTCAAGCACAACAATTTGAAGGATCTGGAGTCGCAGTTAGCTCAGTACCCCAAGTCGACTCCTAAACTGATCGCCTTCGAGTCAGTTTACTCTATGTGTGGATCTGTGGCTCCCATTGAGAAGATTTGCGATTTGGCTAAAAGGTACGGTGCCCTCACCTTCTTGGATGAAGTTCATGCTGTTGGAATGTATGGTCCTCATGGACAGGGTGTAGCTGAGCATTTGGACTTTGATCTGCATTTACAGTCTGGAATCGCCAGTCCTAGCGTGGTGGACAAACGCACCATATTGGATCGTGTCGACATGATTACTGGTACTTGCGGAAAGTCATTTGGTACTGTTGGAGGTTACGTTGCTGGTAGTGCCAACCTAATTGATTGGTTAAGATCCTATGCGCCAGGTTTCATTTTCACTACCACACTTCCTCCTGCTATCATGGCTGGTACAGCCACTTCTGTTCGTATTGTTAGGGCCGACATTGAGGCCCGTATCAAGCAACAGCTTAATACTCGCTACGTCAAAGACTCATTTGAAAACCTTGGTATTCCAGTCATTCCAAACCCAAGTCACATTGTTCCTGTTCTAGTTGGAAATGCTGCAGATGCCAAGAAGGCATCCGATATGTTAATGAACAAACACCGTATTTATGTTCAAGCTATTAACTACCCTACTGTGCCTGTCGGTGAAGAACGACTAAGGATTACTCCTACTCCAGGTCATGGAAAGGAGATTTGTGACCAGCTGATCAGCGCTGTCGACGATGTTTTTACTGAGCTTAATTTACCAAGAATCAACAAATGGCAGTCCCAAGGTGGTCATTGCGGTGTTGGTGATGCTAATTACGTACCAGAACCCAATCTGTGGACTCAGGACCAGCTCAGCTTGACAAACCAAGACTTGCACTCCAATGTGCACAACCCAGTGATTGAGCAGATCGAAACCTCATCAGGAGTCAGATTGTAG

**>PpHEM2**

ATGGTGCATAAGGCTGAATACTTGGACGACCACCCAACTCAGATTTCCAGCATTCTTTCAGGAGGTTACAACCACCCATTACTTCGTGAATGGCAACATGAACGTCAACTCAACAAAAACATGTTCATCTTTCCCCTGTTTGTCACAGATCGACCAGACGAAGAAGAACTTATTCCTAGTCTACCTAATATCAAGAGGTTTGGCGTTAACAAGTTGATTCCTTATGTAGGAGGTTTGGTTTCCAAAGGATTGAGGGCGGTGATCCTATTTGGTGTTCCTCTGAAGCCCGGTGTGAAAGATGAAGAAGGAACGGCCGCTGATGATCCAGAGGGACCTGTTATCCAAGCCATCAAACACTTGAGAAAGAACTTTCCTGACCTGTATATCATCACCGATGTCTGTCTATGTGAGTACACCAGCCATGGACATTGTGGAATACTATATGAGGATGGCACTATCAACAGAGAGCTCTCAGTCCGTCGTATTGCTGCTGTAGCTGTCAAATATGCTCAAGCTGGAGCCAACTCTGTGGCTCCTTCTGATATGACTGACGGCAGAATAAGAGATATTAAAGAAGGCTTACTAAGTGCAGGACTGGCACATAAAACGTTTGTTATGTCCTACGCTGCAAAATTCTCTGGTAATTTGTATGGCCCTTTCAGAGATGCTGCAGGTTCCTGTCCATCTCAAGGGGACAGAAAATGTTACCAGCTTCCTTCTGGAGGAAAAGGGTTGGCCCATCGTGCTCTGATTCGTGATATGAATGAAGGCACTGATGGAATTATTGTCAAACCATCTACATTCTATTTGGACATTGTCGCTGATGCTTATCAGCTTTGTAAAGACTATCCTATCTGCTGTTACCAGGTTTCTGGAGAGTACGCCATGCTACATGCAGCGGCAGAGAAGAATATTGTTGATCTGAAATCAATCGCTTTTGAAGCTCATCAAGGATTCTTGCGGGCTGGAGCTCGTTTAATCATTAGTTACTTTACCCCTGAATTCCTGGAGTGGTTATCTGAATGA

**>PpHEM3**

ATGTTATCTCCAGTATCACAAAACTACCAATCAAGCACAACTCTTTTGACGACAGGCAATACAGCCCACTCAGCTATGCTAGAGAGCTATCAGAACATGAACCAAATCGAACAGAGCGGACCCATTGATTGCAGTTCCTTGAAATTGGGGTCCCGAAAGTCCGCTCTGGCTATAATCCAGGCAGAAATCGTCCGCCAATTGATATTGAAAGAATACCCTGAATTGGAGACGAAGTTGGTCAGTGTGTCCACCCTGGGGGACCAAGTCCAGAATAAAGCACTTTTCACGTTTGGAGGAAAATCTTTGTGGACCAAAGAACTTGAGATGTTGTTGTTGGAGAGTGTGGGAGGATTTGACCAAATAGACATGATTGTACACTCGTTGAAAGACATGCCAACTCATTTACCAGACGAATTTGAGCTGGGTTGCATTATTGAAAGAGAAGACCCTAGAGACGCTTTGGTCGTGCAAGATGGTTTATCTTACAAGTCATTGGCCGACCTTCCAGAGGGAGCTGTGGTCGGTACGTCTTCGGTTAGAAGATCGGCTCAACTACTGAAGAATTTCCCTCATCTGAAATTCAAATCTGTTAGAGGAAACCTTCAGACCAGACTAAGAAAATTAGATGATCCAGATTCCGAGTACTGCTGTCTCCTCCTTGCAGCAGCCGGTTTAATCAGGACAGGCTTACAACACAGAATTTCAATGTATTTGAACGACGATGTGATGTACCACTCCGTCGGACAAGGAGCATTAGGAGTAGAGATCAGAAAAGGTGACCAATTCATGAAAAATATCTGTGAAAAGATTGGGCATAGAACCACCACCCTTCGTTGTCTTGCAGAGAGAGCACTGCTGAGATATCTAGAGGGAGGCTGCTCGGTGCCAATTGGGGTCTCCACTATTTATAGCGAGGATACGAAGGAACTTACCATGAACTCCCTAGTCGTCAGTTGTAACGGTCGTGACTCGGTAACAGAATCAATGACTGAAGTCGTGACTACTGAAGAGCAAGCTGAAGATTTCGGTGAAAGGCTGGCCCAGAAGCTCATAGATCAAGGTGCGAAACGCATTCTTGACGAGATCAACTTCAACAAGATCAAAGAGATTAAGGAAGAGGGTTTACATTAA

**>PpHEM4**

ATGCCAAAAGCCATTCTTCTGAAGAATAAAACTACACCGAAGGATCCTTATCTGGAGAACTTCGTAAGTAGTGGCTACTCGACCGATTTCGTACCACTTTTAGATCATATTCACATGGAGAAATCTGAGATCATCGCATTTCTCAAGACTGACTACTTTTTGCATAAAACTTTGGCGTTTATTATTACGTCCCAAAGAGCTGTAGAAATGCTGAATGAGTGTATGCAAATACTGAGACGTACTGATCCTGAAATTACACAAATCATCTATAGTAAACCTGTCTATACAGTTGGCCCTGCCACCTACAGAATACTTGCGGATGCTGGCTTCGTGGATCTACGAGGCGGAGATAAGGCAGGAAACGGATCCATTCTAGCCCAGATAATTTTGAATGATGACATTTACACTGGAATTGAAGATTCTGACAAGCATATAACGTTTTTCACGGGAGAAACAAGGAGAGACATAATTCCCAAATGTTTACTCTCTAACAACTTTCAACTTTACGAAAAGATTGTCTACAAGACTCTTCCTAGGGATGATATCGTGACTAGATTCAAGTCTGCCGTTGACAGCATCGACCAATCGCAAAGAAGTTCCAGTTGGGTGGTCTTCTTTTCGCCTCAAGGAACAGAGGACATTGTAACGTATCTTCAACACACCAAAGACCAATTTAATATTGCATCTATCGGGCCAACCACAGAAAAATACCTTCTAAGCAAAAACCTGAAACCAAAAGTTGTGGCACCTAAGCCAGAGCCTATCTCTTTACTATTGTCTATACAAAAAGTGCACTAA

**>PpHEM12**

ATGAGTAGATTTCCAGAACTGAAGAATGACCTTATTTTAAGGGCAGCTCGTGGTGAAAAAGTTGAACGTCCCCCAATATGGATTATGAGACAGGCCGGAAGATATCTTCCGGAGTACCATGAGGTCAAAGGAGGTAGGGACTTCTTTGAAACTTGCAGGGATGCTGAGATTGCTTCTGAAATTACTATCCAGCCGATTACGCATTTTGACGGTCTGATCGATGCAGCTATTATCTTCAGTGATATCTTGGTGATTCCTCAAGCTATGGGCATGGAAGTTAAGATGGTGGACAAAGTTGGCCCACAGTTCCCCAATCCGCTAAGAAAACCGTCTGACTTGGATCATTTGAAAAAAGACGTTGACGTTTTGAAGGAACTCGATTGGGCCTTCAAAGCTATCTCATTGACCAGAAAAAAACTCAATGGACGAGTGCCTTTGCTTGGATTTTGTGGTGCTCCTTGGACTCTACTGGTTTATATGACTGAAGGAGGCGGTACCAAGATGTTTCGATTTGCAAAAGAGTGGATCTACAAGTTTACCAAGGAATCTCATCAATTACTCCAACAGATCACTGACGTTGCAGTTGAATTCTTAGCTCAGCAAGTTGTTGCAGGTGCCCAAATGTTACAAGTTTTTGAATCTTGGGGCGGTGAATTGGGGCCTGATGAATTCGATGAGTTTTCTTTGCCTTATTTGAGACAGATTTCCTCTAAACTTCCCCTGAGGTTGAAGGAACTTGGAATCACAGAGAATGTTCCCATAACTGTCTTTGCTAAAGGCTCTTGGTACGCCTTGGAGCAATTGTGCGACAGTGGTTATGATGTTGTCTCGTTGGATTGGTTATTCCGTCCAAGTGATGCTGTCCAGATTGCTAACGGAAGAATCGCATTGCAAGGTAATCTTGACCCTGGAACCATGTACGGCTCCAAAGAAACCATTTCCAAGAAAGTGGACAAAATGATCAAGGGTTTTGGTGGAGGAAAGCAAAACTACATAATTAATTTTGGACACGGCACTCATCCATTCATGGATCCAGAACAGATCAGATGGTTCTTACAAGAATGTCATCGCATTGGATCTCAATAG

**>PpHEM13**

ATGGCCATCGACTCTGATATCAATCTAAGCTCTCCCAATGATTCCATCCGTCAAAGGATGTTCGAGCTTATCCAGCGGAAGCAACTCGAAATTGTCGCTGCATTGGAGGCAATTGAAGGAAACGATACCAAATTTCGTTCTGATTCTTGGGAAAGAGGAGCCGAAGGTGGAGGAGGAAGATCTATGCTTATTCAAGATGGAAGAGTGTTTGAAAAGGCTGGTGTAAATATTTCCAAGGTTCATGGCGTATTGCCTCCTCAAGCTGTGAGCCAGATGAGAAATGACCACTCCAAGCTAGATCTGCCTGCGGGAACCTCTCTGAAGTTCTTTGCCTGTGGGCTTTCGTTGGTCATTCATCCCCATAATCCCCATGCTCCAACTACCCATCTGAATTATCGCTACTTCGAAACTTGGGATGAAACTGGAAAGCCTCACACCTGGTGGTTTGGGGGCGGTGCTGATTTAACGCCTTCGTACCTGTATCCCGAGGATGCCAAGCAATTCCATCAAGCCCATAAGGATGCCCTGGACAAACACGATGTTAGCTTGTACCCGAGATTCAAAAAGTGGTGTGATGAATACTTTCTGATCAAACATCGAAATGAAACTAGAGGTATTGGGGGTATTTTCTTTGATGATTTTGACGAGTTTGATGCTGAGAGGTCCCTGAAGTTGGTTGAAGATTGTTTCAATGCTTTCTTGGAATCTTATCCCGCTATCACTCGAAAAAGGATGGACACCCCTTCAACTGATGCTGAGAAGAACTGGCAACAAATTAGAAGAGGAAGATATGTCGAATTCAACTTAGTATTGGATAGAGGTACTCAATTTGGTTTGAGAACGCCTGGATCTCGTGTTGAAAGTATTTTGATGTCGTTGCCAAGAACAGCTGGTTGGGTCTATGATCATCATCCAGAGCCTGGCTCCAGAGAAGAGGAGTTATTGCAGGTACTACAAAATCCTATTGAATGGGTATGA

**>PpHEM14**

ATGCTGAAAAGTCTTGCACCAAATTCCTCAATTGCCGTTTTAGGTTCAGGGATATCTGGATTGACTTTCAGCTTTTTTTTGAATCGGTTGCGTCCCGATGTTAAGATCCATATCTTTGAAAAATCCAAGCAGGTTGGAGGATGGATCAGATCAGAAGAGCATGAAACTTTTCATTTTGAAAAGGGACCCAGAACTTTGAGAGGCACAAATACGGGTACCTTGATGTTGTTGGATCTTCTTACCAAGATAGGAGCAAATGACAAGGTCCTGGGACTGCACAAAGATTCTCTTGCTAATAAAAAGTATCTGTTGTCCCCGTTCTCAGATGTTCACGGAAACAACGCAAAGCTTCTTCAAGTGCCACAGGATTTCAGCTCTTTTGTAAAGTTCATGTTTGACCCGTTGTCTAAGGATCTCATTCTCGGTCTTTTGAAAGAACCATGGCAACCAAAATTAAAGTATTCAGATGAGTCGGTTGACCATTTTTTCAACAGAAGATTTGCTACCAAACTATCAGAGAATATCGTCAGCGCAATTGTGCATGGAATCTATGCGGGCGACGTGAAGAAGTTAAGTGTGAAAGCCATCTTCCCTAGGCTCCCTGAGATGGAACAGGAAAGTGGCTCTATTATAAGGTATATGATCGCCCAATACAGGACAAAAAAGAACGTCAAACAAAAAGTTGACCCTTTTTTGGCAGATTATGAAAAATTGATCGGTACATCTTTGAGTTTCAAAAATATTTCTTTGTTTCTGAAAAACTTTCCCATGCTGAGTTTTCAGGGTGGACTACAGAAACTTCCCATCTCATTGAAGAACCATTTATCACAGATTGAAAACATCAAGTTTCATTTTGACAGCAAAATCAAAAACATTGCTTTGGAGAGCGGTAAGGTGGCATTGACTGACCATGATCAGGTTTATCTTGTTGACCATGTGAGATCTACCATTAATACCAACGAATTGGCCAAAATCATTTCACCCGTTGTTCCAAGTTCTACTAAGAAAAAATCCGTTTTCAAATCCAAAGCGAATGGCCCAGGGCTGGTCAAATGTTTGAGCTGGCTACACTATACAAATATACTAATGTGCAACATTTATATACCTAAGCACGTCTCAAAATCTATCACCGGATTTGGATACTTGGTTCCTCGATCAATGTCTTCTCAGGCATCCAAACTTCTCGGTGTCATATTTGACTCAGACATCGAGACTGCAATGACTCCTAATTTTACAGAGGCCAACATTACGGCGATAAACAGTAACTCTGCATCTCCCAAGCAACTCCAAAAGTTTTCTGACCAATTCGTCAATAATGATCTCCCTAAATACACCAAGTTGACGCTAATGCTTGGAGGTCATTATCTCAAGTCGGAGGCAGACATGCCCGGTTCCGCAGAGAGTAAACATGCTGTCAAGGCGATTCTGTCAAATCACCTGAATATTGATCTAGATGAGTTTGCATCTTTGCCAGACTTCAAGATGGAAATCACCAAGATCCCCAACTGCATTCCCCAATATGAAGTTGGGTATCTTGATCTCAAGAGAAAGGTTCAGAATGCAGCCTCCAAAGAGTTCAACGACCAAATAAGTTTTGGAGGCATGGCATTTGGTGATGGTGTGGGGATCCCTGACTGTGTCCAGAATGCATTCAAAGATTCGGCTACCCTCAGTGGCATTTAA

**>PpHEM15**

ATGCTTAACCGTCGTTTCCAATCTACCGTGTCCTCGAGTCTGAACAAGGGCACTGGAATAGTGTTCATGAATATGGGTGGTCCCTCCACTGTCAAGGAAACCTATGACTTTTTATTTCGTCTTTTCTCGGACGGAGATTTAATCCCGTTTGGCAGATTTCAGAACATCCTGGCCCGCTTCATTGCAAGTAGAAGAACACCCAAAATTGAATCCTACTACAAAGCTATCGGAGGTGGGTCTCCTATCCGAAAGTGGTCTGAATACCAGAGTTCTAAACTATGTGAAAAATTAGACATTATCAGTCCACAATCGGCTCCTCATAAGCCTTATGTTGCCTTCAGATACGCTAATCCTCTCACTGAAGATACTTTACAAAAGATGAAAAATGATGGAATTACTAAGGCCATTGCCTTTTCTCAATATCCGCAATTTAGTTATTCAACCACCGGATCATCGATTAACGAACTTTACAGGCAATCGAAAATTTTGGACCCTGATCAATCTATTAAATGGACAGTTATAGATCGCTGGCCTGACCACCCAGCCTTAGTTAAAACTTTCGCAGCTCATATCAAAGATACTCTAAACAGATTCAAAACTGAAAATGGACTGACTGACACAAAAGACGTCGTCCTCCAATTCAGTGCTCATTCTTTACCAATGGATATTGTCAATAAAGGAGATTCGTATCCTGCAGAAGTCGCAGCGAGTGTCTTTGCCATTATGAAAGAACTTAACTTCTCAAATCCTTATAAATTAACCTGGCAATCACAGGTTGGCCCAAAGCCTTGGCTGGGTGCTCAAACTGAAAAAATTACCAAGCAGCTAGCATCCAGTGATGTTCCTGGAGTCGTTTTGGTTCCTATTGCCTTTACCTCTGATCATATTGAAACTCTCCATGAACTGGATATTGAACTTATTCAAGAACTACCTAATCCTTCAAAAGTAAAGCGAGTTGAATCGTTGAACGGAGACCAAACTTTCATTGACTCCTTGGCAGAACTAGTGAAGAGTCACATTGATTCGAAGGTTGTATTTTCCAACCAGTTGCCATTGGATTCCATGCTGGGAGTAGTGTCAGATAATTCCCTCACAGATCCAAAAGAGTTTTTCAGAGCCCATTGA
