# Supplementary figures and images for: Optimizing cofactor availability for the production of recombinant heme peroxidase in Pichia pastoris
Source: Microb Cell Fact. 2015 Jan 13;14:4. doi: 10.1186/s12934-014-0187-z (PMC4299804; doi:10.1186/s12934-014-0187-z)

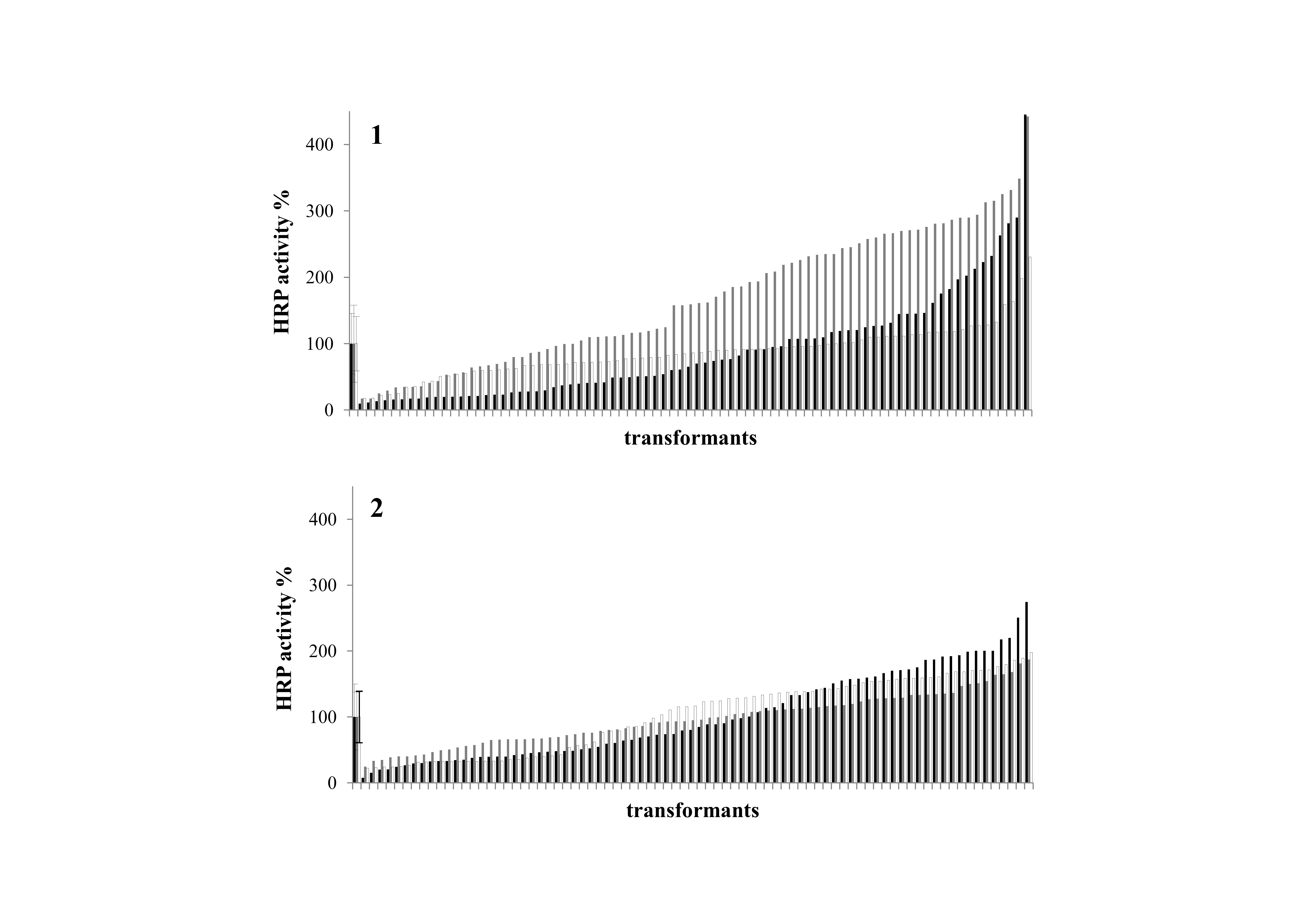

Supplement: Additional file 2: — Co-overexpression of eGFP from P AOX1 . The principial applicability of the employed co-overexpression construct is demonstrated indirectly by eGFP fluorescence of the strains co-overexpressing HRP and eGFP. The first two bars represent the starting strain, PpMutS, and the benchmark strain as controls. Fluorescence was determined at ex/em 488/507 nm, and normalized to the OD600 to account for potential growth differences. [file 12934_2014_187_MOESM2_ESM.jpeg]

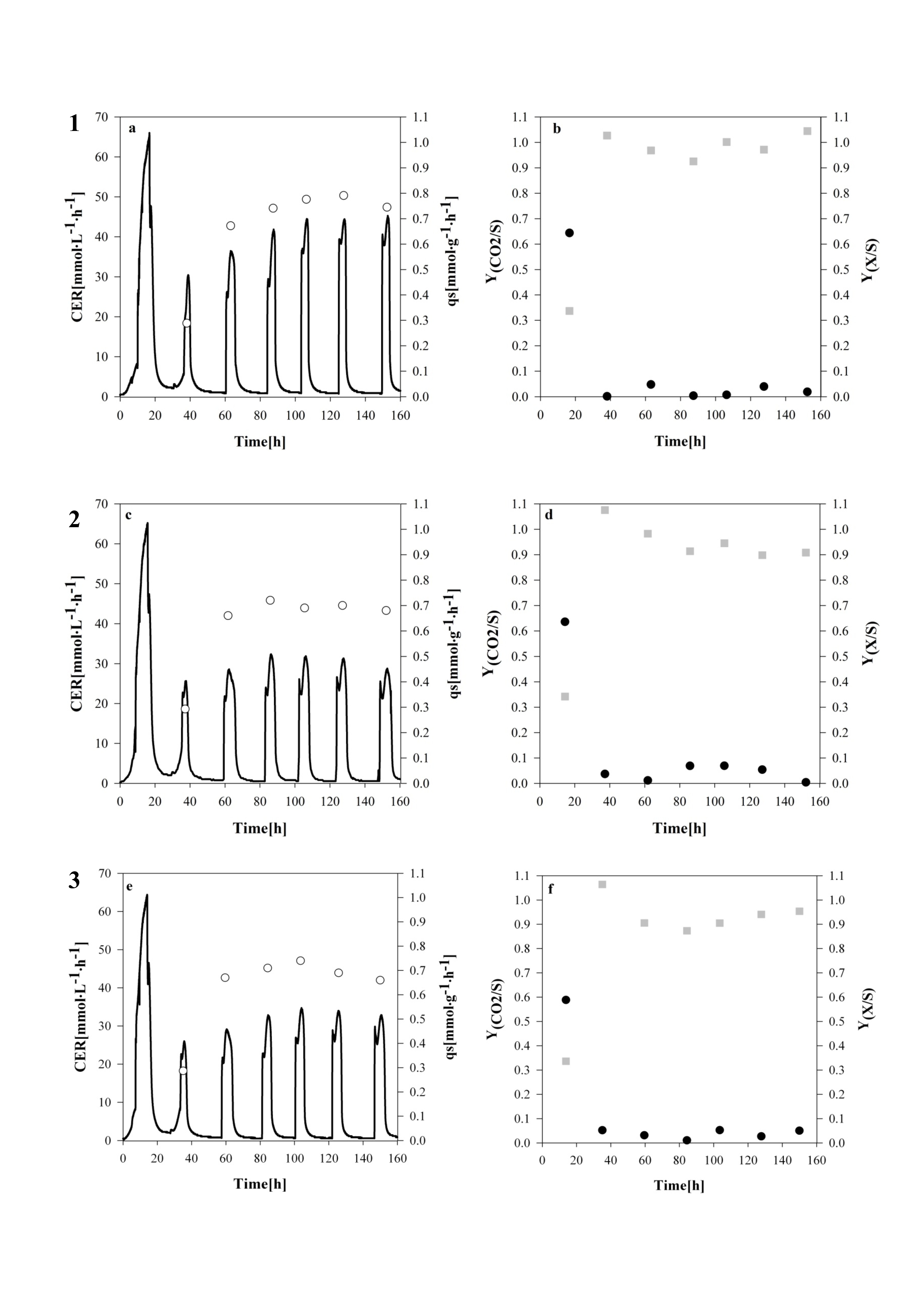

Supplement: Additional file 4: — Batch cultivations of three recombinant P. pastoris strains. 1, benchmark strain; 2, HEM1 strain; 3, HEM3 strain; a/c/e, CER signal (solid line) and specific methanol uptake rate (open circles); b/d/f, carbon dioxide yields (YCO2/S; grey squares) and biomass yields (YX/S; black circles). [file 12934_2014_187_MOESM4_ESM.jpeg]
